# Variation in the Oxytocin Receptor Gene Is Associated with Face Recognition and its Neural Correlates

**DOI:** 10.3389/fnbeh.2016.00178

**Published:** 2016-09-22

**Authors:** Lars Westberg, Susanne Henningsson, Anna Zettergren, Joakim Svärd, Daniel Hovey, Tian Lin, Natalie C. Ebner, Håkan Fischer

**Affiliations:** ^1^Department of Pharmacology, Institute of Neuroscience and Physiology at the Sahlgrenska Academy, University of GothenburgGothenburg, Sweden; ^2^Department of Psychiatry and Neurochemistry, Institute of Neuroscience and Physiology at the Sahlgrenska Academy, University of GothenburgGothenburg, Sweden; ^3^Aging Research Center, Karolinska InstitutetStockholm, Sweden; ^4^Department of Psychology, University of FloridaGainesville, FL, USA; ^5^Department of Aging and Geriatric Research, University of FloridaGainesville, FL, USA; ^6^Department of Psychology, Stockholm UniversityStockholm, Sweden

**Keywords:** oxytocin, social cognition, face recognition, polymorphism, *OXTR*

## Abstract

The ability to recognize faces is crucial for daily social interactions. Recent studies suggest that intranasal oxytocin administration improves social recognition in humans. Oxytocin signaling in the amygdala plays an essential role for social recognition in mice, and oxytocin administration has been shown to influence amygdala activity in humans. It is therefore possible that the effects of oxytocin on human social recognition depend on mechanisms that take place in the amygdala—a central region for memory processing also in humans. Variation in the gene encoding the oxytocin receptor (*OXTR*) has been associated with several aspects of social behavior. The present study examined the potential associations between nine *OXTR* polymorphisms, distributed across the gene, and the ability to recognize faces, as well as face-elicited amygdala activity measured by functional magnetic resonance imaging (fMRI) during incidental encoding of faces. The *OXTR* 3′ polymorphism rs7632287, previously related to social bonding behavior and autism risk, was associated with participants’ ability to recognize faces. Carriers of the GA genotype, associated with enhanced memory, displayed higher amygdala activity during face encoding compared to carriers of the GG genotype. In line with work in rodents, these findings suggest that, in humans, naturally occurring endogenous modulation of OXTR function affects social recognition through an amygdala-dependent mechanism. These findings contribute to the understanding of how oxytocin regulates human social behaviors.

## Introduction

Social recognition refers to the ability to recognize the identity of a previously encountered individual. This ability is essential for successful social interactions. While, in rodents, social recognition is based on olfactory signaling, in humans it is mainly based on visual and auditory cues (Gheusi et al., 1994; Popik and van Ree, 1998; Belin et al., 2011; Peretto et al., 2014). In rodents, the neuropeptide oxytocin is essential for social recognition (Ferguson et al., 2001, 2002). Growing evidence suggests a similar role for oxytocin in humans. Intranasal oxytocin administration has been shown to enhance memory for neutral (Savaskan et al., 2008; Rimmele et al., 2009), happy (Guastella et al., 2008b; Rimmele et al., 2009) and threatening (Savaskan et al., 2008; Rimmele et al., 2009) faces. The findings are however inconsistent; negative findings exist for neutral (Guastella et al., 2008b), happy (Savaskan et al., 2008) and threatening (Guastella et al., 2008b) faces, and oxytocin administration has also been reported to impair face memory (Herzmann et al., 2012).

Oxytocin is a nine-amino-acid-long peptide, with peripheral and central functions. It is synthesized in neurons of the paraventricular and supraoptic nuclei of the hypothalamus and is released through the posterior pituitary gland into the periphery. It is also released into the brain by local dendrites and synapses in regions including the amygdala, the hippocampus, the striatum and the brainstem (Ross and Young, 2009)**.** The effects of oxytocin are mediated by the G-protein-coupled oxytocin receptor (OXTR), which is expressed in different regions in different species in a manner that suggests that its involvement in social attention is conserved through evolution (see Yoshida et al., 2009; Boccia et al., 2013; Freeman et al., 2014). In mice, the involvement of oxytocin in social recognition is mediated through actions in the medial amygdala (Ferguson et al., 2001). In humans, amygdala activation is involved in successful memory encoding (Hamann et al., 1999; Kensinger and Schacter, 2006) and it is also one of several regions processing social information including faces (Bernstein and Yovel, 2015). Whereas convincing evidence of OXTRs in the human amygdala is missing (Loup et al., 1991; Boccia et al., 2013; Freeman et al., 2014), its presence either in the amygdala or in regions tightly coupled to it, is indicated by studies demonstrating an influence of intranasally administered oxytocin on amygdala activity to socially relevant stimuli. There is no consensus regarding the direction of this effect for neutral and happy face stimuli (Domes et al., 2007; Shin et al., 2015) but for threatening social stimuli there appears to be a sex difference such that oxytocin decreases amygdala activity in men (Domes et al., 2007; Kanat et al., 2015) and increases it in women (Domes et al., 2010; Lischke et al., 2012). A recent study however found enhanced amygdala activation to socially relevant threat-associated faces in men only, whereas in women, amygdala activation was found for faces conveying positive social information (Gao et al., 2016).

Taken together, these findings suggest that exogenous pharmacological oxytocin treatment can influence face processing in humans. However, it remains open to what extent endogenous oxytocin regulates the ability to recognize faces. A positive association between variants in oxytocin-related genes, such as the gene *OXTR*, and the ability to recognize faces would support the role of endogenous oxytocin in social recognition in humans. Recent evidence for an association between a single nucleotide polymorphism (SNP) in *OXTR* and face recognition abilities comes from Skuse et al. (2014). Polymorphisms in the *OXTR* gene have also been associated with face-elicited activity in (Tost et al., 2010), and structure of (Inoue et al., 2010), the amygdala, as well as with various social behaviors and risk for disorders related to compromised social cognition (Westberg and Walum, 2014; LoParo and Waldman, 2015).

The current study had two primary aims: to investigate the association between variation in the *OXTR* gene and the ability to recognize neutral faces in healthy adults, and to examine variations in amygdala activity during processing and implicit encoding of faces as a function of *OXTR* genotypes. We hypothesized that carriers of a genotype associated with better social memory would elicit higher amygdala activity during successful face encoding.

## Materials and Methods

### Participants

The original cohort comprised 30 healthy young and 32 healthy older adults (see Ebner et al., 2012 for a detailed description of the sample). Behavioral memory data were lost for two participants due to technical problems with the response pad, and functional magnetic resonance imaging (fMRI) data were lost from two participants due to poor image quality arising from movement artifacts. Genotyping failed for four participants, leaving a sample of 54 participants, 25 young (range 20–31 years, mean ± SD: 25.1 ± 3.4, 12 females) and 29 older (range 65–74 years, mean ± SD: 68.2 ± 2.5, 17 females) adults. The analyses reported in this article refer to this subsample. All participants were Caucasian, as indicated by self-report. They were right-handed native Swedish speakers, had normal or corrected-to-normal vision, had no history of stroke, heart disease, or primary degenerative neurological disorder and were free from blood-thinning medication, as well as from past and present neuropsychiatric diseases, diabetes and neurological disorders. This was assessed by self-reported medical history. Participants also had no contraindications to MRI. For older adults, a radiologist screened a T1-weighted and a T2-weighted image and ruled out abnormal levels of atrophy or lesions. All participants provided informed consent. The study was approved by the regional ethical review board of Stockholm. Informed consent was obtained from all participants in accordance with the declaration of Helsinki.

### Face Encoding and Recognition Paradigm

The face encoding and recognition paradigm was the first test of a larger cognitive and socioemotional test battery that participants underwent during fMRI. In a single fMRI incidental encoding run (8.4 min), participants passively viewed color photographs of 48 individual faces displaying neutral expressions. The photographs were presented in pseudo-random order and consisted of equal numbers of male and female, young and older faces. All faces were taken from the FACES database (Ebner et al., 2012). Each face was presented for 3500 ms, followed by presentation of a fixation cross for 3000–4000 ms (jittered). One third of the trials were pseudo-randomly intermixed low-level baseline trials, where three x’s were presented on the screen. No more than two faces of the same age or gender, no more than three faces and no more than two low-level baseline events followed in sequence. During encoding the participants were unaware of the following recognition session.

A retention interval of approximately 8–10 min, during which structural scans were taken, separated encoding from the two fMRI recognition runs. During the recognition phase, participants saw 96 faces, 48 target and 48 distracter faces, and were instructed to make old–new judgments, as accurately and quickly as possible, while the face was presented on the screen. The pseudo-random order of presentation for the target faces, randomly interspersed with distracter faces, was different from that at encoding. The presentation mode was otherwise identical to that at encoding, including also the low-level baseline trials where participants chose response buttons themselves. After this task the participants performed one other additional task in the scanner, lasting 22 min.

As the primary behavioral outcome measure, we calculated dprime, reflecting the participants’ ability to correctly recognize target faces (correctly targeted as old) controlled for false recognition of distracter faces (commission errors). Specifically, dprime is determined as *Z*(hit frequency) − *Z*(false alarm frequency), where *Z* is the inverse of the cumulative distribution function of the Gaussian distribution (Stanislaw and Todorov, 1999).

### Brain Imaging

fMRI was performed on a 3T Siemens Magnetom Trio Tim scanner. The procedures for image acquisition and preprocessing have been described previously (Lovén et al., 2014). SPM8 was used for data preprocessing and analysis. Based on previous literature regarding the influence of oxytocin on amygdala activity and the importance of the amygdala for memory, we focused on this region of interest (ROI). The amygdala was defined by the WFU-Pickatlas (Maldjian et al., 2003). MarsBaR (Brett et al., 2002) was used to extract from the first-level design, each individual’s average parameter estimates from the left and right amygdala respectively, and for the contrasts: faces minus low-level baseline and Remembered faces minus low-level baseline. Although this procedure excludes subregion analysis of the amygdala activity, this was not considered a serious caveat since the 3T resolution together with the spatial smoothing (8 mm) in the data analysis does not allow for reliable conclusions regarding the involvement of separate subregions within the small structure of amygdala.

### Genotyping

Thirteen *OXTR* SNPs (rs75775, rs2270465, rs2268498, rs2301261*, rs4564970*, rs4686302*, rs237897, rs53576, rs2254298*, rs2268493, rs237887, rs1042778, rs7632287) were genotyped by LGC Genomics^1^ using KASPar methodology (see Table 1). The SNPs were distributed throughout the gene and had previously been shown to be associated with social behaviors (Westberg and Walum, 2014; LoParo and Waldman, 2015). Four SNPs were excluded from the analyses due to minor allele frequencies <10%, i.e., rs4564970, rs2301261, rs4686302, rs2254298, indicated above with an asterisk. Three of the four were in high linkage disequilibrium (LD) and part of the same haplotype block (Campbell et al., 2011).

**Table 1:** **Association analysis results for the oxytocin receptor (*OXTR*) polymorphisms and face recognition (dprime)**.

SNP	Position	MAF	*F*	*df*	*p*-value
rs75775	5′	0.21	0.09	2,49	0.9^#^
rs2270465	5′	0.28	0.16	2,50	0.86^#^
rs2268498	5′	0.50	0.14	2,48	0.87
rs237897	intron 3	0.44	0.94	2,51	0.40
rs53576	intron 3	0.34	0.45	2,50	0.64^#^
rs2268493	intron 3	0.38	0.84	2,49	0.44
rs237887	intron 3	0.38	0.72	2,49	0.49
rs1042778	3′	0.47	0.69	2,50	0.51
rs7632287	3′	0.25	4.75	2,50	0.013^#^

*MAF; minor allele frequency. ^#^<5 carriers for less frequent homozygote*.

### Group Statistical Analysis

We used SPSS general linear models procedure to conduct analyses of variance (ANOVAs) for each of the SNPs with respect to dprime. The SNPs were treated as random factors. None displayed a significant deviation from homogeneity of variances as indicated by non-significant Levene’s tests. Sex and age were likewise controlled for by adding them as random factors to the general linear model. *Post hoc t*-tests were used to further specify the effects. Polymorphisms that displayed a relation with dprime were also tested for potential associations with face-elicited amygdala activity during encoding. Results at *p* < 0.05 were considered statistically significant. Because of the potential dependence between polymorphisms due to high LD, the corrected *p*-value for the ANOVAs was determined by permutation testing, using R software and permuting the order of the phenotype values (dprime) ten thousand times and determining the number of times one of the nine polymorphisms randomly resulted in a significant *p*-value.

## Results

**Figure 1:**
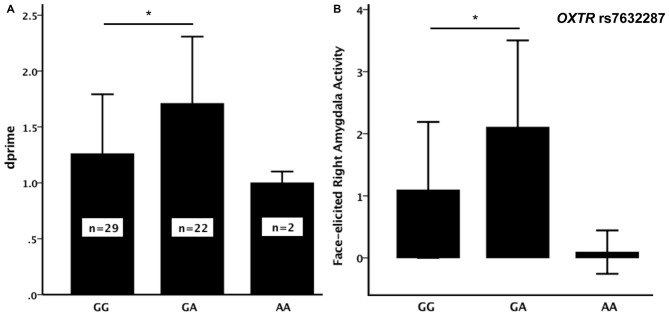
**(A)** Face recognition performance (dprime, mean ± SD) and **(B)** face-elicited right amygdala activity (mean (over participants) average parameter estimates for the amygdala as defined by the WFU-Pickatlas ± SD) for the three genotypes of the *oxytocin receptor (OXTR)* rs7632287 single nucleotide polymorphism (SNP). There was a significant difference (*) in dprime (*p* = 0.007, *t*_49_ = 2.84) and face-elicited right amygdala activity (*p* = 0.006, *t*_49_ = 2.90) between GG and GA carriers.

Out of the nine *OXTR* SNPs that were analyzed in relation to face recognition ability, the rs7632287 displayed a nominally significant association with dprime (*p* = 0.013, *F*_(2,50)_ = 4.7, Table 1, Figure 1A) that did not survive correction for multiple testing (corrected *p* = 0.06). *Post hoc t*-tests showed that the association was due to enhanced face recognition skills in GA carriers as compared to GG carriers (*p* = 0.007, *t*_49_ = 2.8, Figure 1A). Controlling the significant difference in face recognition between GA and GG carriers for sex (*p* = 0.006, *p*_sex_ = 0.42) and age (*p* = 0.015, *p*_age_ = 0.25), showed that neither contributed significantly to face recognition abilities, and that the results for the *OXTR* polymorphism did not change markedly.

The sample only contained two carriers of the AA genotype. Since the mean dprime values for the AA and GA genotypes were not similar (Figure 1A), we did not consider it suitable to pool the two genotypes for the subsequent analyses. There was also no significant difference in dprime between carriers of the AA genotype and any of the other two genotype groups (*p* > 0.3). The analysis of the brain imaging results therefore focused on testing whether the difference in face memory displayed between the GA and GG genotypes could be explained by differences in face-elicited amygdala activity between the genotype groups. During incidental encoding of faces, the activity in the right amygdala was significantly larger for carriers of the GA genotype than for carriers of the GG genotype (*p* = 0.006, *t*_49_ = 2.9, Figure 1B).

There was a correlation between dprime and face-elicited right (*p* = 0.04, Pearson correlation= 0.28), but not left (*p* = 0.12), amygdala activity. To elucidate whether the difference in face memory between GA and GG carriers may be related to the difference in face-elicited right amygdala activity as suggested by the association and correlation, we also tested, using a paired-samples *t*-test, whether the amygdala activity to those faces that were later remembered was greater than that to faces in general. The faces that were later remembered did elicit larger activity (*p* = 0.00014, *t*_53_ = 4.8). Moreover, the activity of the right amygdala, elicited by faces that were later remembered, was also larger in GA- than in GG-carriers of the rs7632287 (*p* = 0.001, *t*_49_ = 3.7).

## Discussion

Our study suggests an association in healthy individuals between the *OXTR* SNP rs7632287 and the ability to correctly recognize faces. Carriers of the GA genotype, associated with better memory for faces, displayed more activity in the right amygdala during passive viewing (incidental encoding) of faces, compared to GG carriers. Since the right amygdala activity correlated with face recognition memory, it is possible that the increased amygdala activity observed in carriers of this genotype at least partly explains their superior memory. This interpretation is supported by the finding that faces that were later remembered elicited more right amygdala activity than faces in general.

The findings are in line with rodent work showing that oxytocin acts in the amygdala during encoding of social olfactory information (Ferguson et al., 2002). Although there are extensive differences across species regarding social perception pathways and the distribution of OXTRs, our results support the view that the role of oxytocin for encoding of social information has been conserved through evolution (Freeman et al., 2014; Skuse et al., 2014; Wigton et al., 2015). Several mechanisms have been suggested for oxytocin’s influence on different aspects of social cognition in humans. There is evidence that oxytocin increases overall gaze time toward faces, particularly toward the eye region of faces (Guastella et al., 2008a; Bartz et al., 2011)—a visual scan pattern that is critical for making inferences about face stimuli, and that may facilitate encoding of facial identity. These findings are in line with the general hypothesis that oxytocin alters the perceptional salience of social and socioemotional stimuli (Bartz et al., 2011; Gao et al., 2016). To examine the difference between oxytocin’s effects on the processing and memory of faces expressing different emotions, future studies should include happy and threatening faces in addition to neutral. Since oxytocin administration has been shown to influence face judgments of valence and attractivity (Theodoridou et al., 2009; Shahrestani et al., 2013; Ellingsen et al., 2014), these factors should also be taken into account as potential mediators of the effect; it is possible that the difference in face memory observed between the genotypes is related to differences in face judgments.

There are no established functional polymorphisms in *OXTR*, and several SNPs in the gene have therefore been investigated in relation to social behaviors and social deficits. The rs7632287 SNP, associated with face recognition and amygdala activity in the current study, has previously been associated with childhood social problems in girls and pair-bonding in women (Walum et al., 2012), social responsiveness in children with autism spectrum disorder (Harrison et al., 2015) and antisocial behavior in boys (Hovey et al., 2016), in a manner suggesting that the A-allele, or more specifically, the AA genotype, is associated with impaired social cognition. In the current study, a limitation was that there were too few AA-carriers to be able to draw any conclusions regarding this genotype group (Figure 1A). This could be avoided by selecting participants on genotype information in order to ensure that all genotype groups are sufficiently represented in the sample. Moreover, from these previous findings it might be expected that the A-allele, including also the GA genotype, would be associated with *compromised* social cognition. Instead our findings show that the carriers of the GA genotype had *superior* mean face recognition abilities compared to GG carriers. In line with the direction of the current association, two studies have reported an association between the G allele and autism spectrum disorder (Tansey et al., 2010; Campbell et al., 2011), and a meta-analysis showed the association between rs7632287 and autism to be the most robust amongst *OXTR* polymorphisms (LoParo and Waldman, 2015). Thus, our findings of associations between rs7632287 and face recognition and correlating amygdala activity add support to the emerging view that rs7632287, or any polymorphism in LD with rs7632287, may affect OXTR function.

Of note, the rs7632287 SNP was not genotyped by Skuse et al. (2014), which is currently the only study investigating *OXTR* SNPs in relation to social recognition abilities in humans. In a large sample of relatives to patients with autism spectrum disorder, they reported an association between rs237887 and social recognition. We did not observe an association for this polymorphism. Inconsistencies in findings across studies may be due to differences in inclusion criteria of participants, ethnicity, age, the social recognition paradigms used and statistical power. Notably, the 3′ rs7632287 has been reported neither to be in high LD with the intron 3 rs237887, nor with the more adjacent rs1042778, also genotyped in this study (Campbell et al., 2011; Johansson et al., 2012).

A serious caveat of the current study is that the sample size was small. The power thus did not allow for additional tests in other ROIs related to face and memory processing. Considering the small effects by which individual polymorphisms may influence complex traits (Rietveld et al., 2014), our choice to genotype several *OXTR* SNPs in this low-powered sample provided us with small chances of revealing associations that survive stringent correction for multiple testing. Indeed, the initial association between rs7632287 and face recognition did not survive rigorous correction for multiple testing. Thus, replication of our findings in independent, larger samples is needed. Although there is emerging evidence for an influence of the rs7632287 or an adjacent SNP on oxytocinergic function, this should not prevent future studies from investigating genetic variation throughout the gene. The often used approach of investigating single, possibly non-functional, intronic variants is not likely to provide knowledge about the full contribution of *OXTR* to inter-individual variation in social behaviors—a view supported by recent meta-analyses (Bakermans-Kranenburg and van Ijzendoorn, 2014; Li et al., 2015).

To conclude, our results suggest a moderating role of a 3′ *OXTR* SNP on the ability to encode faces in humans, with a functional site in the right amygdala. In line with work in rodents, these findings suggest that naturally occurring endogenous modulation of OXTR function in humans affects social recognition through an amygdala-dependent mechanism. These findings contribute to the understanding of how oxytocin regulates human social behaviors.

## Author Contributions

Study concept and design: LW. Acquisition, analysis, or interpretation of data: All authors. Drafting of the manuscript: SH and LW. Critical revision of the manuscript for important intellectual content: All authors. Final approval of the version to be published: All authors. Agreement to be accountable for all aspects of the work in ensuring that questions related to the accuracy or integrity of any part of the work are appropriately investigated and resolved: All authors.

## Conflict of Interest Statement

The authors declare that the research was conducted in the absence of any commercial or financial relationships that could be construed as a potential conflict of interest.

## Footnotes

^1^www.lgcgenomics.com
